# Antimicrobial Drug–Resistant Shiga Toxin–Producing *Escherichia coli* Infections, Michigan, USA

**DOI:** 10.3201/eid2309.170523

**Published:** 2017-09

**Authors:** Sanjana Mukherjee, Rebekah E. Mosci, Chase M. Anderson, Brian A. Snyder, James Collins, James T. Rudrik, Shannon D. Manning

**Affiliations:** Michigan State University, East Lansing, Michigan, USA (S. Mukherjee, R.E. Mosci, C.M. Anderson, B.A. Snyder, S.D. Manning);; Michigan Department of Health and Human Services, Lansing (J. Collins, J.T. Rudrik)

**Keywords:** *Escherichia coli*, Shiga toxin–producing *E. coli*, STEC, antibiotic resistance, epidemiology, risk factors, antimicrobial resistance, bacteria, Michigan, United States

## Abstract

High frequencies of antimicrobial drug resistance were observed in O157 and non-O157 Shiga toxin–producing *E. coli* strains recovered from patients in Michigan during 2010–2014. Resistance was more common in non-O157 strains and independently associated with hospitalization, indicating that resistance could contribute to more severe disease outcomes.

Shiga toxin–producing *Escherichia coli* (STEC) contributes to 265,000 cases of foodborne illness annually in the United States (*1*). Most infections are caused by O157 strains; however, non-O157 STEC infections have increased (*2*). Antimicrobial drug resistance among STEC has been reported (*3*–*5*) but is probably underestimated. Given the importance of resistance in *E. coli* pathotypes, we sought to determine the prevalence of resistant STEC infections and assess the effects of resistance on disease.

We obtained 358 STEC isolates from the Michigan Department of Health and Human Services (MDHHS) Reference Laboratory (Lansing, MI, USA), collected during 2010–2014. Of these, 14 were outbreak associated. We examined 1 strain per outbreak using protocols approved by Michigan State University (MSU; Lansing, MI, USA; IRB #10-736SM) and MDHHS (842-PHALAB). Overall, 31 (8.8%) strains (23 non-O157, 8 O157) were resistant to antimicrobial drugs (Table). Resistance to ampicillin (7.4%) was most common, followed by trimethoprim/sulfamethoxazole (SXT) (4.0%) and ciprofloxacin (0.3%). Compared with national rates, resistance to ampicillin and SXT was higher, but not significantly different, for O157 isolates from Michigan (Technical Appendix Figure 1) (*6*). One strain was resistant to all drugs, and all resistant strains had high MICs (ampicillin, >64 μg/mL; ciprofloxacin, >32 μg/mL; SXT, in 1:19 ratio, >32/608 μg/mL). Notably, resistance was twice as common for non-O157 (11.1%) than for O157 (5.5%) strains. O111 strains (n = 7) had significantly higher resistance frequencies (24.1%) than other non-O157 serogroups (p = 0.03). We found variation by year and season; resistance frequencies were highest in 2012 (Technical Appendix, Figure 2) and during winter/spring (Technical Appendix Table 1), but neither trend was significant. We also observed a strong but nonsignificant association between resistance and hospitalization but no association for urban versus rural residence (*7*) or county after stratifying by prescription rates (*8*) in the univariate analyses.

**Table:** Antimicrobial drug resistance in 353 clinical Shiga toxin–producing *Escherichia coli* isolates, by serotype, Michigan, USA, 2010–2014*

**Serotype**	**No. isolates**	**No. (%) isolates**			
		Any resistance	Ampicillin resistance	Ciprofloxacin resistance	SXT resistance
**O157**	146	8 (5.5)	7 (4.8)	0 (0)	5 (3.4)
**Non-O157**	207	23 (11.1)	19 (9.2)	1 (0.5)	9 (4.3)
O26	53	4 (7.6)	4 (7.6)	0 (0)	1 (1.9)
O45	50	6 (12.0)	5 (10.0)	0 (0)	2 (4.0)
O103	75	6 (8.0)	5 (6.7)	1 (1.3)	4 (5.3)
O111	29	7 (24.1)	5 (17.2)	0 (0)	2 (6.9)

*We tested 358 isolates by disk diffusion for resistance to ampicillin (10 μg in disk), SXT (25 μg in disk), and ciprofloxacin (5 μg in disk). MICs were determined by using Etest. Strains were classified as resistant or susceptible according to Clinical Laboratory Standards Institute guidelines; *E. coli* ATCC 25922 was used as a control. Five isolates had unknown serotypes and were excluded from analysis. Isolate numbers for individual antibiotics do not always add up to the total number of isolates with any resistance because some isolates were resistant to >1 drug. SXT, trimethoprim/ sulfamethoxazole.

We conducted a multivariate analysis using logistic regression, with hospitalization as the dependent variable; we included variables with significant (p<0.05) and strong (p<0.20) associations from the univariate analysis as independent variables. Forward selection indicated that hospitalized patients were more likely to have resistant infections (odds ratio [OR] 2.4, 95% CI 1.00–5.82) and less likely to have non-O157 infections (OR 0.4, 95% CI 0.21–0.61) (Technical Appendix Table 2), suggesting that resistant infections or O157 infections may cause more severe clinical outcomes. Patients >18 years of age, women, and patients with bloody diarrhea were also more likely to be hospitalized. 

Although we found no significant difference by *stx* profile, strains possessing *stx*1 only were more commonly resistant than strains with *stx*2 alone (p = 0.27 by Fisher exact test). All 23 (100%) resistant non-O157 STEC and 1 (12.5%) resistant O157 strain had *stx*1 only. Strains positive for *eae* were less likely to be resistant (n = 27; 8.4%) than *eae*-negative strains (n = 4; 23.5%); this nonsignificant difference (p = 0.07 by Fisher exact test) could be due to small sample sizes. All 8 resistant O157 strains and 18 (78.3%) of 23 resistant non-O157 strains had *eae*, demonstrating correlations between virulence genes and serogroups.

Overall, we detected a high frequency of resistance among non-O157 STEC (11.2%), similar to findings from Mexico (15%), although we evaluated fewer drugs (*5*). Resistance to ciprofloxacin was low despite its routine use for treating enteric infections, perhaps because resistance development in *E. coli* requires multiple mutations (*9*). Resistance frequencies in STEC were low relative to other *E. coli* pathotypes such as extraintestinal *E. coli*, which may be attributable to differences in the source of the infections (*3*). 

The higher O157 resistance frequencies in Michigan than nationwide indicate that selection pressures vary by location and source. Although we observed no difference in resistance frequencies for counties with high versus low antimicrobial drug prescription rates (*8*), we have not investigated selection pressures from drug use in farm environments that may affect resistance emergence in Michigan. Approximately 12 × 10^6^ kg of antimicrobial drugs are administered to food animals annually in the United States; roughly 61% of these are medically relevant. Higher resistance frequencies in winter/spring (12.2%) than summer/fall (7.5%) could be attributed to variation in prescription rates by season (*10*).

Because Michigan is not part of the Centers for Disease Control and Prevention Foodborne Diseases Active Surveillance Network and resistance in STEC has not been widely researched, data about the prevalence and impact of resistance are lacking. This study detected a high frequency of STEC resistance to antimicrobial drugs commonly used in human and veterinary medicine, particularly for non-O157 serotypes, which have increased in frequency (*2*). Monitoring resistance in STEC is essential because of the risk of transmitting resistant strains from food animals to humans and the high likelihood of horizontal transfer of resistance genes from STEC to other pathogens. Routine monitoring can uncover new treatment approaches and guide development of strategies for controlling emergence and spread of resistance in STEC and other *E. coli* pathotypes.

Technical Appendix Additional information for analysis of antimicrobial drug resistance in Shiga toxin–producing *Escherichia coli* (STEC) in Michigan, 2010–2014.
