# Supplementary material for: Antimicrobial Drug–Resistant Shiga Toxin–Producing Escherichia coli Infections, Michigan, USA
Source: Emerg Infect Dis. 2017 Sep;23(9):1609–11. doi: 10.3201/eid2309.170523 (PMC5572870; doi:10.3201/eid2309.170523)
Supplement: Technical Appendix — Additional information for analysis of antimicrobial drug resistance in Shiga toxin–producing Escherichia coli (STEC) in Michigan, 2010–2014. [file 17-0523-Techapp-s1.pdf]

Article DOI: https://doi.org/10.3201/eid2309.170523

Antimicrobial Drug–Resistant Shiga Toxin–Producing *Escherichia coli* Infections, Michigan, USA

Technical Appendix

Technical Appendix Table 1. Univariate analysis highlighting factors associated with antibiotic resistance in 358 clinical Shiga toxin–producing *Escherichia coli* (STEC) in Michigan, 2010–2014

Characteristic	Total strains*	No (%) resistant	OR (95% CI†)	p-value‡
Pathogen factors				
Serotype				
O157	146	8 (5.5)	1.0	–
Non-O157	207	23 (11.1)	2.2 (0.94–4.97)	0.066
stx profile				
*stx*1	205	25 (12.2)	1.9 (0.72–5.28)	0.18
*stx*2	75	5 (6.7)	1.0	–
stx1,*stx2*	77	2 (2.6)	0.3 (0.07–1.99)	0.27
eae presence				
Yes	323	27 (8.4)	0.3 (0.10–1.04)	0.05
No	18	4 (22.2)	1.0	–
Outbreak associated				
Yes	14	1 (7.1)	0.8 (0.10–6.14)	0.81
No	344	31 (9.0)	1.0	–
Demographics and other factors				
Residence				
Urban	153	13 (8.5)	0.9 (0.43–1.90)	0.80
Rural	205	19 (9.3)	1.0	–
Age, y				
0–18	154	12 (7.8)	1.0	–
19–64	172	17 (9.9)	1.3 (0.60–2.81)	0.51
$\geq$65	32	3 (9.4)	1.2 (0.32–4.61)	0.76
Sex				
Male	173	14 (8.1)	1.0	–
Female	185	18 (9.7)	1.2 (0.59–2.54)	0.59
Antimicrobial-drug prescription rates by county				
High	109	13 (11.9)	1.6 (0.78–3.45)	0.19
Low	249	19 (7.6)	1.0	–
Season				
Winter and spring	115	14 (12.2)	1.7 (0.83–3.62)	0.14
Summer and fall	243	18 (7.4)	1.0	–
Clinical factors				
Abdominal pain				
Yes	279	27 (9.7)	1.4 (0.48–4.23)	0.53
No	57	4 (7.0)	1.0	–
Body ache				
Yes	55	7 (12.7)	1.6 (0.64–3.83)	0.33
No	281	24 (8.5)	1.0	–
Bloody diarrhea				
Yes	232	21 (9.1)	0.9 (0.42–2.06)	0.87
No	104	10 (9.6)	1.0	–
Hemolytic uremic syndrome (HUS)				
Yes	6	0 (0)	–	1.0
No	331	31 (9.4)	–	
Hospitalization				
Yes	106	13 (12.3)	1.7 (0.80–3.61)	0.16
No	237	18 (7.6)	1.0	–

*Epidemiological data and case information were retrieved from the Michigan Disease Surveillance System (MDSS). SAS 9.3 (SAS Institute, Cary, NC) and Epi Info™ 7 (CDC) were used for statistical analyses. Depending on the variable examined, the number of isolates do not add up to the total (n=358) because of missing data.

Characteristic	Total strains*	No (%) resistant	OR (95% CI†)	p-value‡

†95% confidence interval (CI) for odds ratio (OR)
‡*p*-value was calculated by Chi-square test,; Fisher exact test was used for variables <u><</u>5 in at least 1 cell

Technical Appendix Table 2. Univariate and multivariate analyses to identify factors associated with hospitalization

Characteristic	Total strains*	No (%) hospitalized	OR (95% CI)†	p-value‡
Serotype				
O157	138	63 (45.7%)	1.0	–
Non-O157	200	42 (21.0%)	0.3 (0.20–0.51)	<0.0001
stx profile				
*stx*1	198	43 (21.7%)	0.3 (0.18–0.58)	<0.0001
*stx*2	72	33 (45.8%)	1.0	–
stx1,*stx2*	72	30 (41.7%)	1.7 (0.86–3.20)	0.13
eae presence				
Yes	310	92 (29.7)	0.5 (0.20–1.50)	0.23
No	16	7 (43.8)	1.0	–
Outbreak associated				
Yes	14	7 (50.0)	2.5 (0.79–6.80)	0.11
No	329	99 (30.1)	1.0	–
Antimicrobial drug resistant isolate				
Yes	31	13 (41.9)	1.7 (0.80–3.61)	0.16
No	312	93 (29.8)	1.0	–
Sex				
Male	166	39 (23.5)	1.0	–
Female	177	67 (37.9)	2.0 (1.24–3.17)	0.004
Age, y				
0–18	145	35 (24.1)	1.0	–
19–64	167	56 (33.5)	1.6 (0.96–2.61)	0.07
<u>></u>65	31	15 (48.4)	2.9 (1.32– 6.56	0.007
Abdominal pain				
Yes	277	95 (34.3)	2.2 (1.08–4.41)	0.03
No	57	11 (19.3)	1.0	–
Body ache				
Yes	55	20 (36.4)	1.3 (0.70–2.35)	0.42
No	279	86 (30.8)	1.0	–
Bloody diarrhea				
Yes	230	91 (39.6)	3.9 (2.12–7.13)	<0.0001
No	104	15 (14.4)	1.0	–
Hemolytic uremic syndrome (HUS)				
Yes	5	4 (80.0)	9.0 (0.99–81.45)	0.02
No	328	101 (30.8)	1.0	–

		Multivariate logistic regression associations §	
Characteristic	OR	95% CI ¶	*p*-value
Sex: F	1.9	1.15–3.32	0.02
Age, y: 18	1.9	1.15–3.28	0.014
Serogroup: non-O157	0.4	0.21–0.61	0.0002
Antimicrobial drug resistant isolate: Yes	2.4	1.00–5.82	0.05
Bloody diarrhea: Yes	3.9	1.99–7.65	<0.0001

*Depending on the variable examined, the number of isolates do not add up to the total (n=358) because of missing data. All 6 HUS cases had O157 strains with *eae*, though 3 had *stx1,stx2* and the other 3 had *stx2* infections
†95% confidence interval (CI) for odds ratio (OR)
‡*p*-value was calculated by Chi-square test; Fisher exact test was used for variables <u><</u>5 in at least 1 cell.
§Logistic regression was performed using forward selection while controlling for variables that yielded significant (P≤0.05) and strong (P≤0.20) associations with hospitalization in the univariate analysis. The model was adjusted for age, sex, serogroup, *stx* profile, outbreak status, resistance, HUS, and bloody diarrhea. Only those variables yielding significant associations are presented; Hosmer and Lemeshow Goodness-of-Fit test (P= 0.73). All variables were tested for collinearity.
¶Wald 95% confidence intervals (CI)

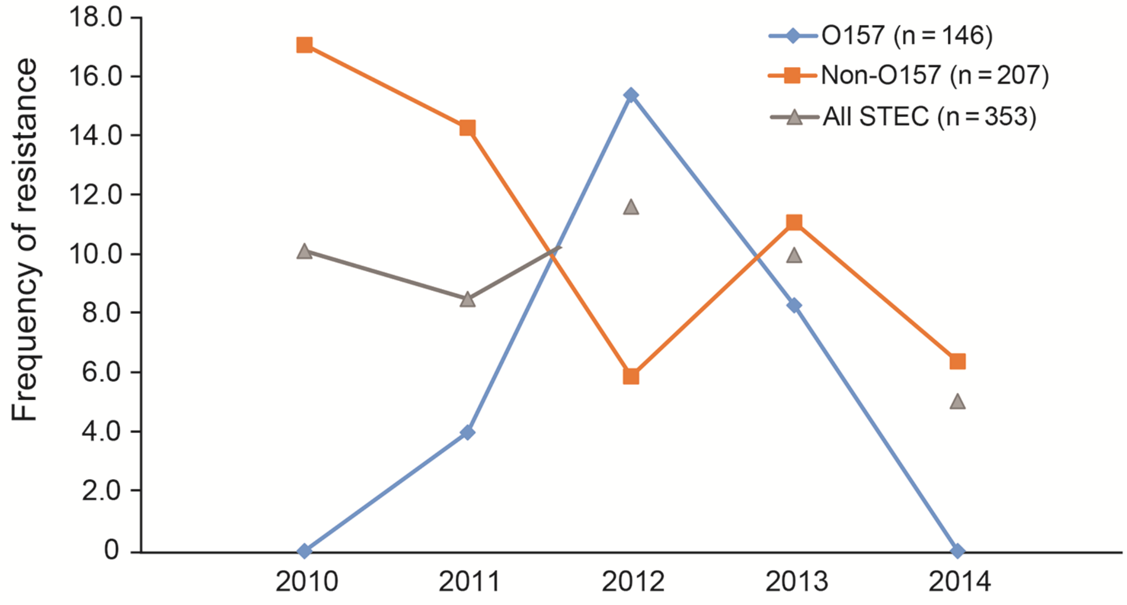

Technical Appendix Figure 1. Frequency of any resistance to ampicillin, ciprofloxacin and trimethoprim-sulfamethoxazole among O157 and non-O157 Shiga toxin producing *E. coli* (STEC) isolates recovered from patients in Michigan, 2010–2014

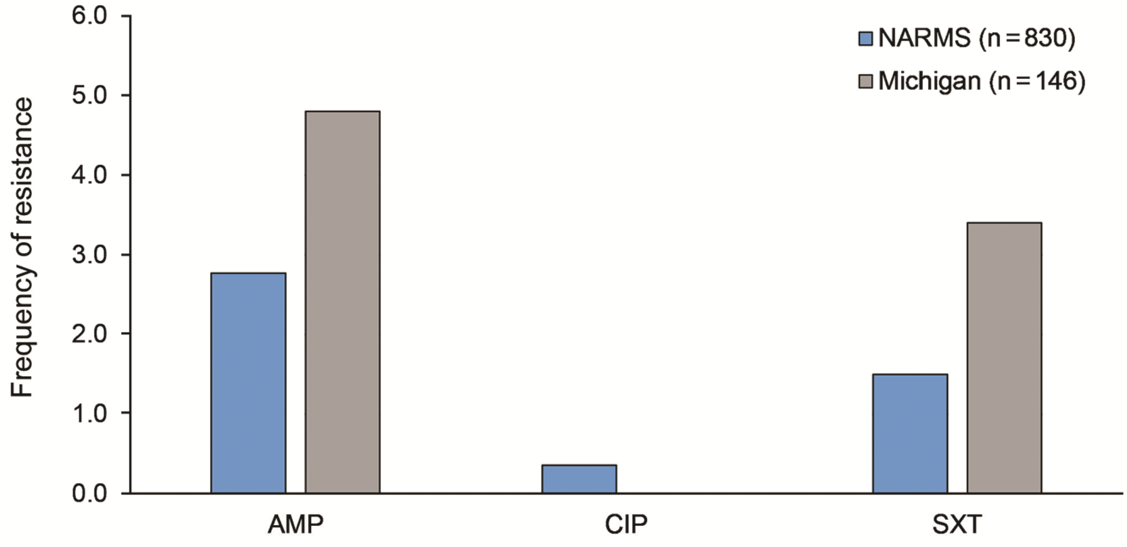

Technical Appendix Figure 2. Frequency of resistance to various antimicrobials among STEC O157 isolates in Michigan compared to those reported by the National Antimicrobial Resistance Monitoring System (NARMS), 2010-2014. Abbreviation: AMP, ampicillin; CIP, ciprofloxacin; SXT, trimethoprim-sulfamethoxazole
